# Fine particulate matter exposure aggravates ischemic injury via NLRP3 inflammasome activation and pyroptosis

**DOI:** 10.1111/cns.13837

**Published:** 2022-04-10

**Authors:** Li Gao, Jie‐Xing Qin, Jian‐Quan Shi, Teng Jiang, Fei Wang, Chong Xie, Qing Gao, Nan Zhi, Qing Dong, Yang‐Tai Guan

**Affiliations:** ^1^ Department of Neurology Ren Ji Hospital Shanghai Jiao Tong University School of Medicine Shanghai China; ^2^ Department of Neurology Nanjing First Hospital Nanjing Medical University Nanjing China

**Keywords:** fine particulate matter (PM2.5), ischemic stroke, NLRP3 inflammasome, pyroptosis, reactive oxygen species (ROS)

## Abstract

**Aims:**

Accumulating evidence has suggested that airborne fine particulate matter (PM2.5) exposure is associated with an increased risk of ischemic stroke. However, the underlying mechanisms have not been fully elucidated. In this study, we aim to investigate the role and mechanisms of NLRP3 inflammasome and pyroptosis in ischemic stroke after PM2.5 exposure.

**Methods:**

The BV‐2 and HMC‐3 microglial cell lines were established and subjected to oxygen–glucose deprivation and reoxygenation (OGD/R) with or without PM2.5 exposure. We used the CCK‐8 assay to explore the effects of PM2.5 on cell viability of BV‐2 and HMC‐3 cells. Then, the effects of PM2.5 exposure on NLRP3 inflammasome and pyroptosis following OGD/R were detected by western blotting, ELISA, and the confocal immunofluorescence staining. Afterwards, NLRP3 was knocked down to further validate the effects of PM2.5 on cell viability, NLRP3 inflammasome activation, and pyroptosis after OGD/R in HMC‐3 cells. Finally, the intracellular reactive oxygen species (ROS) was measured and the ROS inhibitor N‐acetyl‐L‐cysteine (NAC) was used to investigate whether ROS was required for PM2.5‐induced NLRP3 inflammasome activation and pyroptosis under ischemic conditions.

**Results:**

We found that PM2.5 exposure decreased the viability of BV‐2 and HMC‐3 cells in a dose‐ and time‐dependent manner under ischemic conditions. Furthermore, PM2.5 exposure aggravated NLRP3 inflammasome activation and pyroptosis after OGD/R, as indicated by an increased expression of NLRP3, ASC, pro‐caspase‐1, Caspase‐1, GSDMD, and GSDMD‐N; increased production of IL‐1β and IL‐18; and enhanced Caspase‐1 activity and SYTOX green uptake. However, shRNA NLRP3 treatment attenuated the effects of PM2.5 on cell viability, NLRP3 inflammasome activation, and pyroptosis. Moreover, we observed that PM2.5 exposure increased the production of intracellular ROS following OGD/R, while inhibiting ROS production with NAC partially attenuated PM2.5‐induced NLRP3 inflammasome activation and pyroptosis under ischemic conditions.

**Conclusion:**

These results suggested that PM2.5 exposure triggered the activation of NLRP3 inflammasome and pyroptosis under ischemic conditions, which may be mediated by increased ROS production after ischemic stroke. These findings may provide a more enhanced understanding of the interplay between PM2.5 and neuroinflammation and cell death, and reveal a novel mechanism of PM2.5‐mediated toxic effects after ischemic stroke.

## Graphical abstract

PM2.5 exposure triggers the activation of NLRP3 inflammasome and pyroptosis under ischemic conditions, which was mediated by an increased ROS production after ischemic stroke.

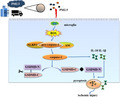

## 1 INTRODUCTION

Stroke is the major cause of death and long‐term disability worldwide, and ischemic stroke accounts for approximately 80% of stroke cases. In addition to the well‐known risk factors for stroke (e.g., hypertension, hyperlipidemia, diabetes mellitus, and smoking), environmental risk factors, such as air pollution, are of increasing interest.^1^, ^2^ Airborne fine particulate matter (PM2.5, aerodynamic diameter < 2.5 μm) is the main component of air pollution and mainly composed of compounds of both organic and inorganic, including sulfates, carbon, nitrates, hydrogen ions, ammonium, lipopolysaccharides (LPS), metals, and water. It has been reported to play a significant role in the development of ischemic stroke.^3^, ^4^ Some epidemiologic studies have demonstrated that both short‐term and long‐term PM2.5 exposures are associated with a higher risk of ischemic stroke, especially with stroke characterized by large artery atherosclerosis (LAA) and small vessel occlusion.^5^, ^6^, ^7^ A recent large cohort study provided further evidence that PM2.5 exposure may be associated with ischemic stroke in individuals with prevalent atrial fibrillation.^8^ In addition, high exposure to particulate matter (PM) before stroke may be independently associated with 3‐month mortality and cerebral edema after intravenous thrombolysis.^9^ However, the mechanisms by which PM2.5 exposure aggravates ischemic injury remain unclear.

In recent decades, a number of studies have demonstrated that innate immunity and inflammatory responses are involved in ischemic brain injury.^10^, ^11^ As a pivotal component of innate immunity, the nucleotide‐binding oligomerization domain‐like receptor pyrin domain‐containing protein 3 (NLRP3) inflammasome is thought to play a crucial role in the pathophysiology of ischemic stroke.^12^ It is a cytoplasmic multiprotein complex mainly comprised of the sensor protein NLRP3, the adapter protein apoptosis‐associated speck‐like protein containing CARD (ASC), and the cysteine protease Caspase‐1.^13^ Recent findings have revealed that the NLRP3 inflammasome is abundantly expressed in brain, especially in microglia, to create a platform for regulating the secretion of interleukin‐1β (IL‐1β) and IL‐18 and engage in immune defenses and inflammatory reactions.^14^, ^15^ It can be activated by a variety of stimuli, such as microbes,^16^ extracellular toxins,^17^ reactive oxygen species (ROS), and urate crystals.^18^, ^19^ Upon detection of certain stimuli, NLRP3 oligomerizes and interacts with ASC to recruit the inactive zymogen pro‐caspase‐1 and then autocleaves into active Caspase‐1 to trigger the maturation of IL‐1β and IL‐18. Furthermore, activated Caspase‐1 can cleave gasdermin D (GSDMD) to initiate a novel form of inflammatory cell death called pyroptosis that is morphologically different from apoptosis.^20^ GSDMD contains approximately 480 amino acids, and the gasdermin‐N domain (GSDMD‐N) has a novel membrane pore‐forming activity, inducing cell swelling and membrane rupture through the release of intracellular contents and proinflammatory mediators.^21^ Accumulating evidence has indicated that the NLRP3 inflammasome and pyroptosis participate in detecting cellular damage and mediating inflammatory responses and cell death following cerebral ischemia. Blocking or inhibiting the NLRP3 inflammasome may offer substantial promise to salvage neurological function during ischemic stroke.^22^, ^23^, ^24^

To date, an increasing number of studies have proven that PM2.5 can induce an inflammatory response, which acts as a major driving force for the development and aggravation of many diseases and causes numerous adverse health effects.^25^, ^26^ Furthermore, recent findings suggest that PM2.5 may trigger NLRP3 inflammasome activation, contributing to the development of lung fibrosis, cardiovascular diseases, and Alzheimer's disease (AD).^27^, ^28^, ^29^ However, whether the NLRP3 inflammasome and pyroptosis are involved in the inflammatory response in PM2.5‐induced ischemic injury has not been elucidated. In this study, we aim to investigate the role and mechanisms of NLRP3 inflammasome and pyroptosis in ischemic stroke after PM2.5 exposure. To do this, we first chose two microglial cell lines and determined the effect of different concentrations of PM2.5 on cell viability with an in vitro oxygen–glucose deprivation and reoxygenation (OGD/R) model. Then, we assessed the effect of PM2.5 exposure on NLRP3 inflammasome activation and pyroptosis following OGD/R. Finally, we explored the mechanism of NLRP3 inflammasome activation and pyroptosis under ischemic conditions after PM2.5 exposure.

## 2 MATERIALS AND METHODS

### 2.1 Source of PM2.5

The PM2.5 (SRM 1649b‐Urban Dust) was purchased from the National Institute of Standards and Technology (NIST; Gaithersburg, MD, USA) and suspended in sterile phosphate buffer solution (PBS) for use in the experiments.

### 2.2 Oxygen–glucose deprivation and reoxygenation (OGD/R) model and PM2.5 exposure

The mouse microglial cell line BV‐2 was purchased from the Shanghai Chinese Academy of Sciences. The human microglial cell line HMC‐3 was purchased from the Shanghai FuHeng Biology (Shanghai, China). The cells were cultured in Dulbecco's modified Eagle's medium (DMEM; HyClone, Logan, UT, USA) containing 10% fetal bovine serum (FBS; HyClone) and 1% penicillin/streptomycin solution (Gibco‐BRL, Grand Island, NY, USA). Both cell lines were incubated in a humidified atmosphere containing 5% CO_2_ and 95% air at 37℃.

OGD/R was induced as previously described.^30^ The cells were rinsed twice with PBS, then incubated in glucose‐free DMEM without FBS in an aerobic chamber (Thermo Fisher Scientific, Waltham, MA, USA). After 4 h of OGD, the medium was replaced with normal medium containing 10% FBS, and the cells were transferred to an incubator under normoxic conditions for five consecutive days. The control cells were treated similarly but not exposed to OGD.

To generate a PM2.5 exposure model, PM2.5 was suspended and sonicated at final concentrations of 25 and 50 μg/ml, which were chosen based on the published literature.^31^ Then, both the cells were exposed to PM2.5 extracts for 6 h before OGD. To treat cells with both the ROS inhibitor N‐acetyl‐L‐cysteine (NAC; Sigma‐Aldrich, St. Louis, USA) and PM2.5, NAC was dissolved in PBS and added to the cultured cells at a final concentration of 5 mM for 2 h before PM2.5 exposure or OGD. The vehicle group was treated with the same amount of PBS.

### 2.3 Cell viability assay

The Cell Counting Kit‐8 (CCK‐8) assay (Dojindo, Kumamoto, Japan) was performed to assess the cell proliferation according to the manufacturer's instructions. The cells were seeded in 96‐well culture plates at a density of 5 × 10^3^ cells/ml in 100 μl culture medium per well. The CCK‐8 solution (1:10) was added to each well at the 24‐, 48‐,72‐,96‐, and 120‐h time points. After 2‐h darkroom incubation at 37°C, the optical density of each well was analyzed at 450 nm with a microplate reader (BioTek Elx800; Winooski, VT, USA).

### 2.4 Knockdown of NLRP3 with shRNAs

For NLRP3 knockdown, three NLRP3‐shRNAs with the following sequences were used: 5′‐GGATCTTCGCTGCGATCAACA‐3′ (NLRP3‐KD1), 5′‐GGTGTACGTCTTCTTCCTTTC‐3′ (NLRP3‐KD2), and 5′‐GCAAGATCTCTCAGCAAATCA‐3′) (NLRP3‐KD3). The knockdown lentiviruses and a negative control (NC) lentivirus were constructed by HanYin Biotech (Shanghai, China). Both the recombinant lentivirus and NC lentivirus were prepared and titered to 10^9^ TU/ml. The BV‐2 and HMC‐3 cells were seeded in 6‐well plates at a density of 2 × 10^5^ cells per well and infected with the same titer of virus with 8 µg/ml Polybrene^®^ (HanYin, Biotech, Shanghai, China) the following day. After 48 h, the cells were harvested and the knockdown efficiency was confirmed by quantitative real‐time PCR (qRT‐PCR). Then, cells exhibiting stable knockdown of NLRP3 were subjected to OGD/R alone, or OGD/R together with PM2.5 exposure.

### 2.5 RNA extraction and quantitative real‐time PCR (qRT‐PCR)

Total RNA was extracted using TRIzol reagent (Invitrogen, Carlsbad, CA, USA). The PrimeScript^®^ RT Reagent Kit (Takara, Bio Inc., Kyoto, Japan) was used to obtain cDNA. Quantitative real‐time PCR was performed using a 7500 Fast Real‐Time PCR System (Applied Biosystems, Carlsbad, CA, USA), and qRT‐PCR was performed with 2 × SYBR Green Gene Expression PCR Master Mix. The following primers were used (5′‐3′):

NLRP3‐F: CACACGACTGCGTCTCATCAAG

NLRP3‐R: GAACACCACGGTGTGCACAG

GAPDH‐F: GTCTCCTCTGACTTCAACAGCG

GAPDH‐R: ACCACCCTGTTGCTGTAGCCAA

### 2.6 Western blotting

The cells were lysed with radioimmunoprecipitation assay (RIPA) buffer (Beyotime, Shanghai, China) containing protease inhibitors (Roche, Complete Mini, Basel, Switzerland). A total of 30–50 μg protein lysate was subjected to 8%–12% SDS‐PAGE, transferred to PVDF membranes, and blocked with 10% nonfat dry milk in Tris‐HCL buffer saline (TBS, pH 7.4) containing 0.1% Tween‐20 (TBST) for 1 h at room temperature. Then, the membranes were incubated with primary antibodies against NLRP3 (1:1000; Novus), ASC (1:1000; Abcam), pro‐caspase‐1 (1:1000; Abcam), caspase‐1 (1:1000; Novus), GSDMD (1:1000; Novus), and GSDMD‐N (1:1000; CST). After being washed in TBST, the membranes were incubated with the corresponding secondary antibody for 2 h at room temperature. Finally, the immunoblots were visualized using enhanced chemiluminescence on the ChemiDoc XRS system and the band densities were normalized to the loading control β‐actin (1:1000; CST).

### 2.7 Enzyme‐linked immunosorbent assay (ELISA)

The total protein concentration was quantitated by a bicinchoninic acid assay kit (Beyotime, Shanghai, China). The protein levels of the inflammatory cytokines IL‐1β and IL‐18 were quantified using specific ELISA kits for IL‐1β (R&D Systems, Minneapolis, MN, USA) and IL‐18 (R&D Systems, Minneapolis), according to the manufacturer's instructions.

### 2.8 Caspase‐1 activity assay

The activity of Caspase‐1 was assayed using the Caspase‐1 Activity Assay Kit (Beyotime, Shanghai, China) according to the manufacturer's instructions. The samples were analyzed, and the absorbance was measured at a wavelength of 405 nm.

### 2.9 ROS production assay

The generation of intracellular ROS was measured using a dichlorofluorescein diacetate (DCFH‐DA) detection kit after the cells were seeded in 6‐well plates in accordance with the manufacturer's instructions (Beyotime, Shanghai). The cells were harvested and incubated with 10 μM/L DCFH‐DA at 37°C for 20 min in darkness. Then, DCF‐derived fluorescence was measured by a BioTek Synergy 2 microplate reader (BioTek Instruments, Winooski, VT, USA) at an excitation wavelength of 488 nm and an emission wavelength of 535 nm.

### 2.10 SYTOX green acid staining

SYTOX green acid staining was performed to assess pyroptosis induced by OGD/R and PM2.5 exposure as described before.^32^ Microplates were treated with 0.05 mg/mL poly‐L‐lysine (Solarbio Life Sciences, Beijing, China) before HMC‐3 cells were seeded. Then, the cells were seeded in the 96‐well plates and SYTOX green acid staining solution was prepared by diluting stock solution (Invitrogen, Carlsbad, CA, USA) at 1:30000 (167 nM) in phosphate‐free buffer. SYTOX and DAPI were added to cover the cells for 15 min in the dark. The cells were then washed three times in phosphate‐free buffer and quickly imaged using a confocal microscope (Leica, Wetzlar, Germany).

### 2.11 Statistical analysis

All statistical analyses were performed using GraphPad Prism 8.0. The data are presented as the mean ± SD. Unless otherwise stated, all quantitative statistical analyses were performed in a blinded manner. Student's *t*‐test (two‐tailed) or the Mann–Whitney test was used to determine differences between two groups according to their Gaussian or non‐Gaussian statistical distribution. For comparisons of three or more groups, one‐way analysis of variance (ANOVA) followed by the Student–Newman–Keuls test was used. Repeated measures were analyzed by two‐way repeated‐measures ANOVA. Differences were considered significant at *p* < 0.05.

## 3 RESULTS

### 3.1 PM2.5 exposure induced the decrease in cell viability under ischemic conditions

**FIGURE 1:**
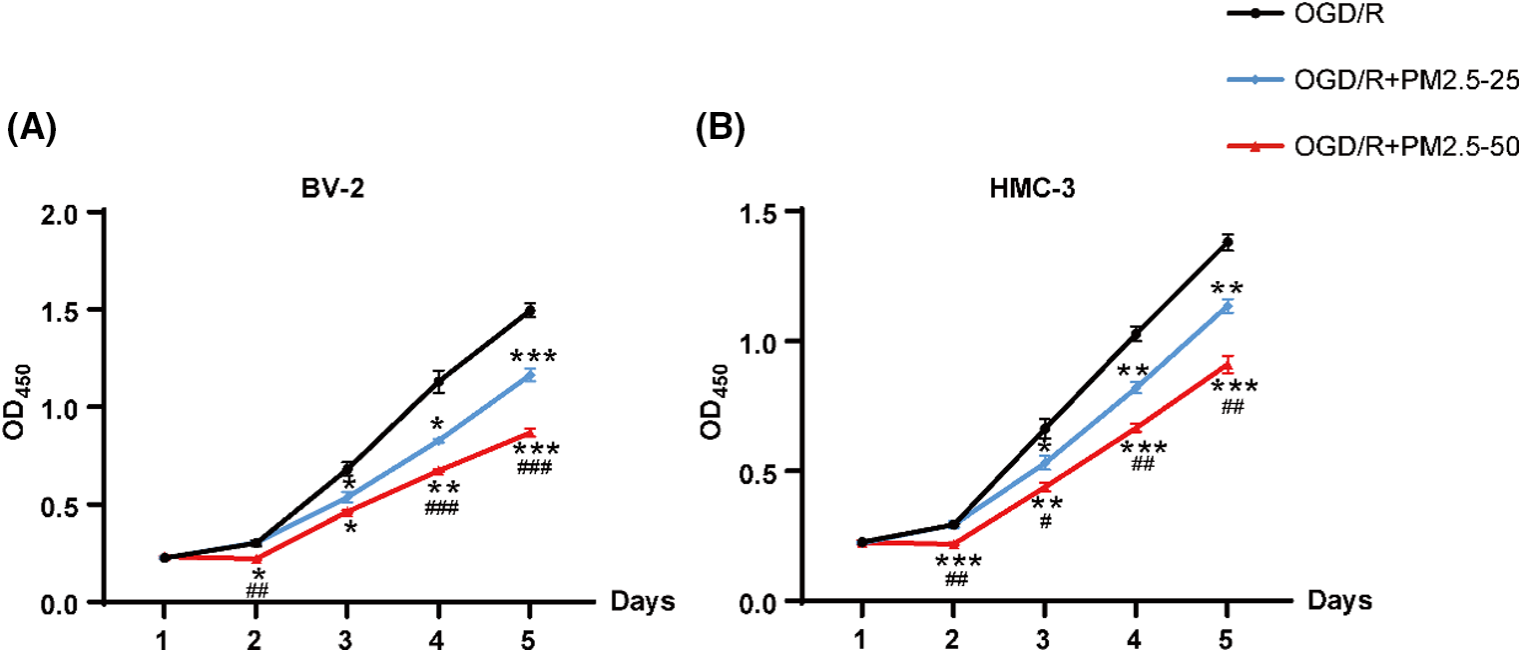
CCK‐8 assay was performed to investigate the effects of PM2.5 on cell viability in BV‐2 and HMC‐3 microglial cells under ischemic conditions. (A) The cell viability curve of BV‐2 cells subjected to OGD/R alone or together with PM2.5 exposure at 25 and 50 μg/ml, respectively. (B) The cell viability curve of HMC‐3 cells subjected to OGD/R alone or together with PM2.5 exposure at 25 and 50 μg/ml, respectively (*p < 0.05, **p < 0.01, ***p < 0.001 vs. OGD/R; *^#^p* < 0.05, *^##^p* < 0.01, *^###^p* < 0.001 vs. OGD/R+PM2.5–25; *n* = 3). OGD/R, oxygen–glucose deprivation/reoxygenation; OGD/R+PM2.5‐25, OGD/R and PM2.5 exposure at 25 μg/ml; OGD/R+PM2.5‐50, OGD/R and PM2.5 exposure at 50 μg/ml

The CCK‐8 assay was carried out to explore the effects of PM2.5 on BV‐2 and HMC‐3 microglial cell viability. As shown in Figure 1, the proliferation of both BV‐2 and HMC‐3 cells decreased gradually and significantly from Day 3 to Day 5 after PM2.5 exposure at a concentration of 25 μg/ml (Figure 1A,B). Furthermore, exposure to a higher concentration of 50 μg/ml PM2.5 more markedly decreased the proliferation of both cell lines from Day 2 to Day 5, especially in HMC‐3 cells. In addition, when compared to the 25 μg/ml PM2.5‐exposed group, the proliferation of both cell lines decreased significantly in the 50 μg/ml PM2.5‐exposed group on different days. These results indicated that PM2.5 exposure inhibited cell viability in a time‐ and dose‐dependent manner under ischemic conditions.

### 3.2 NLRP3 knockdown attenuated the effects of PM2.5 on cell viability following OGD/R

To explore the effects of NLRP3 on cell viability after PM2.5 exposure under ischemic stress, we constructed NLRP3 knockdown BV‐2 and HMC‐3 cell lines. The knockdown efficiency was assessed through qRT‐PCR (Figure S1). Three shRNA sequences were used to knock down NLRP3 in both BV‐2 and HMC‐3 cells, and we chose to use two shRNA sequences (NLRP3‐KD1 and NLRP3‐KD3), which had better knockdown efficiency for the CCK‐8 assay.

**FIGURE 2:**
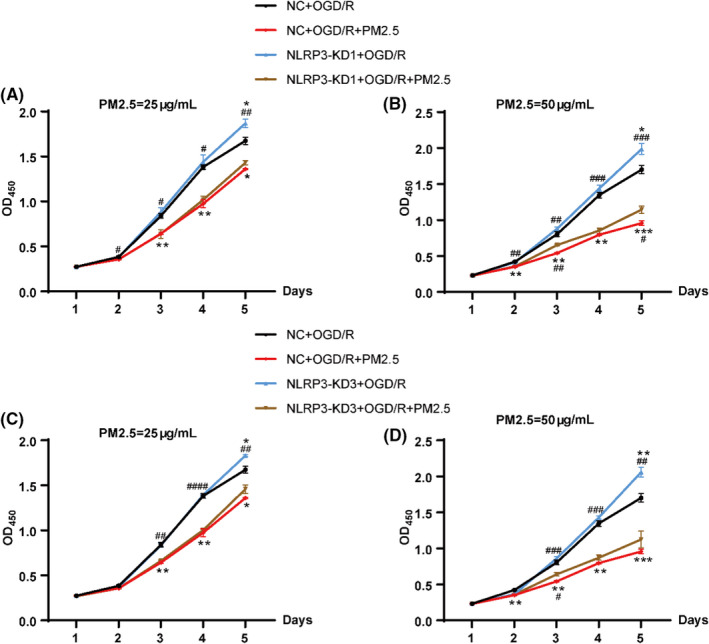
NLRP3 knockdown partially mitigated the effects of PM2.5 on cell viability in BV‐2 microglial cells following OGD/R. (A, B) After treatment with NLRP3‐NC or NLRP3‐KD1, the cell viability of BV‐2 cells was detected in NC+OGD/R, NC+OGD/R+PM2.5, NLRP3‐KD1+OGD/R, and NLRP3‐KD1+OGD/R+PM2.5 groups. The exposure concentrations of PM2.5 were 25 μg/ml (A) and 50 μg/ml (B), respectively (*p < 0.05, **p < 0.01, ***p < 0.001 vs. NC+OGD/R; *^#^p* < 0.05, *^##^p* < 0.01, *^###^p* < 0.001 vs. NLRP3‐KD1+OGD/R+PM2.5; *n* = 3). (C, D) After treatment with NLRP3‐NC or NLRP3‐KD3, the cell viability of BV‐2 cells was detected in NC+OGD/R, NC+OGD/R+PM2.5, NLRP3‐KD3+OGD/R, and NLRP3‐KD3+OGD/R+PM2.5 groups. The exposure concentrations of PM2.5 were 25 μg/ml (C) and 50 μg/ml (D), respectively (*p < 0.05, **p < 0.01, ***p < 0.001 vs. NC+OGD/R; *^#^p* < 0.05, *^##^p* < 0.01, *^###^p* < 0.001, *^####^p* < 0.0001 vs. NLRP3‐KD3+OGD/R+PM2.5; *n* = 3)

BV‐2 cells were divided into 6 groups: the NC+OGD/R, NC+OGD/R+PM2.5, NLRP3‐KD1+OGD/R, NLRP3‐KD1+OGD/R+PM2.5, NLRP3‐KD3+OGD/R, and NLRP3‐KD3+OGD/R+PM2.5. The cells were exposed to PM2.5 at a concentration of 25 or 50 μg/ml. According to the cell viability curve, PM2.5 exposure decreased the cell proliferation following OGD/R, especially at the concentration of 50 μg/ml (Figure 2A–D). However, the inhibitory effects of PM2.5 on cell proliferation were significantly attenuated at Day 3 and Day 5 after NLRP3‐KD1 treatment in the 50 μg/ml PM2.5‐exposed group (Figure 2B). Additionally, these effects also can be observed after NLRP3‐KD3 treatment at Day 3 when exposed to 50 μg/ml PM2.5 (Figure 2D). Consistent with previous studies, we also found the NLRP3 knockdown by both NLRP3 shRNA sequences increased cell proliferation when compared to the NC+OGD groups at Day 5 (Figure 2A–D).^22^, ^24^ Interestingly, we observed that PM2.5 exposure at 25 and 50 μg/ml can still decrease cell proliferation time‐dependently even though NLRP3 was knocked down under ischemic conditions (Figure 2A–D).

**FIGURE 3:**
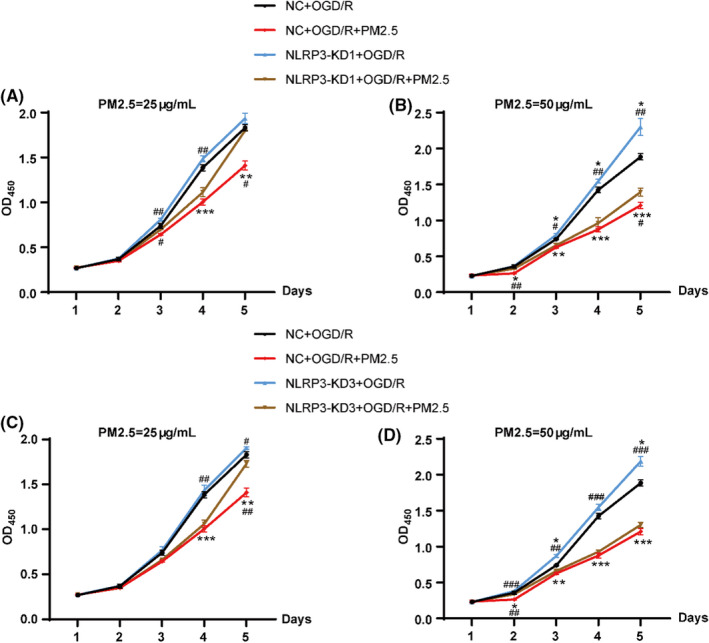
NLRP3 knockdown partially attenuated the effects of PM2.5 on cell viability in HMC‐3 microglial cells under ischemic conditions. (A, B) The cell viability curve of HMC‐3 cells in NC+OGD/R, NC+OGD/R+PM2.5, NLRP3‐KD1+OGD/R, and NLRP3‐KD1+OGD/R+PM2.5 groups. The exposure concentrations of PM2.5 were 25 μg/ml (A) and 50 μg/ml (B), respectively (*p<0.05, **p<0.01, ***p<0.001 vs. NC+OGD/R; *^#^p* < 0.05, *^##^p* < 0.01 vs. NLRP3‐KD1+OGD/R+PM2.5; *n* = 3). (C, D) The cell viability curve of HMC‐3 cells in NC+OGD/R, NC+OGD/R+PM2.5, NLRP3‐KD3+OGD/R, and NLRP3‐KD3+OGD/R+PM2.5 groups. The exposure concentrations of PM2.5 were 25 μg/ml (c) and 50 μg/ml (d), respectively (*p < 0.05, **p < 0.01, ***p < 0.001 vs. NC+OGD/R; *^#^p* < 0.05, *^##^p* < 0.01, *^###^p* < 0.001 vs. NLRP3‐KD3+OGD/R+PM2.5; *n* = 3)

HMC‐3 cells were divided into six groups as well and subjected to the same protocol. As shown in Figure 3, PM2.5 at a concentration of 25 and 50 μg/ml decreased the cell proliferation (Figure 3A–D). The knockdown of NLRP3 with both shRNA sequences partially attenuated the inhibitory effects of PM2.5 on different days, especially at Day 5 in both 25 and 50 μg/ml PM2.5‐exposed groups (Figure 3A–C). Consistently, NLRP3 depletion increased cell proliferation when compared to the NC+OGD groups (Figure 3B,D). In addition, we also observed that when compared with NLRP3‐KD1+OGD/R group, exposure to PM2.5 at both concentrations inhibited cell proliferation on different days, even though NLRP3 was knocked down (Figure 3A–D).

Taken together, the above data further suggested that PM2.5 exposure decreased cell proliferation under ischemic stress, while NLRP3 depletion by two shRNA sequences partially attenuated the inhibitory effects of PM2.5 on cell proliferation at different time points. Considering the pronounced effects of higher concentration of PM2.5 and NLRP3‐KD1, we exposed the HMC‐3 cells to 50 μg/ml PM2.5 for five consecutive days to further explore the relationship between PM2.5 and the NLRP3 inflammasome under ischemic conditions in the following experiments.

### 3.3 PM2.5 exposure promoted NLRP3 inflammasome activation

**FIGURE 4:**
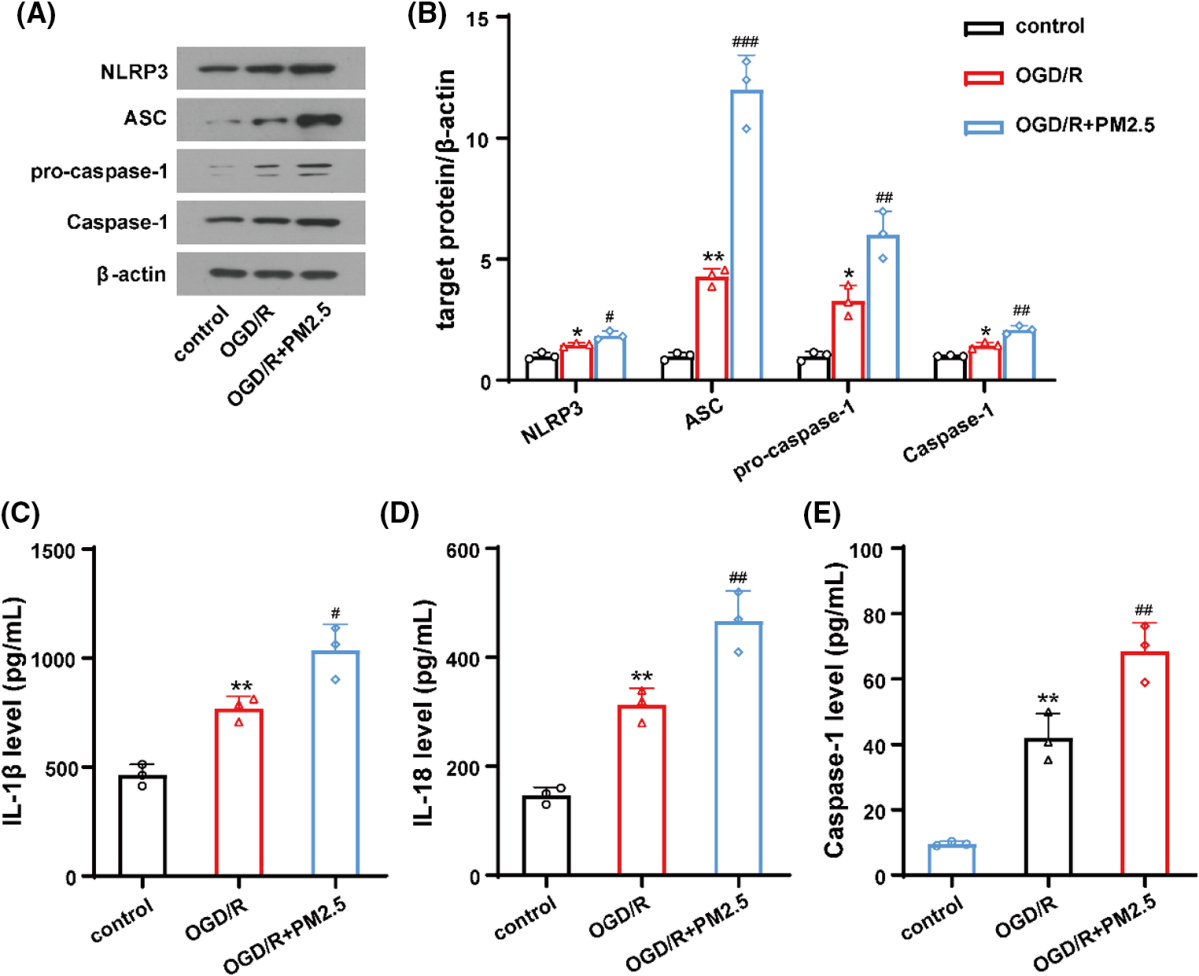
PM2.5 exposure induced NLRP3 inflammasome activation following OGD/R. (A, B) The expressions of NLRP3, ASC, pro‐caspase‐1, and Caspase‐1 in control, OGD/R, and OGD/R+PM2.5 groups. (C, D) The ELISA showed the production of inflammatory cytokines IL‐1β and IL‐18 in three groups. (E) Caspase‐1 activity was assessed after PM2.5 exposure under OGD/R state (*p < 0.05, **p < 0.01 vs. control; *^#^p* < 0.05, *^##^p* < 0.01, *^###^p* < 0.001 vs. OGD/R; *n* = 3)

To determine the effects of PM2.5 on NLRP3 inflammasome activation in HMC‐3 cells under ischemic state, we detected the expression of NLRP3 inflammasome components after the cells subjected to OGD/R and PM2.5 exposure at Day 5. As shown in Figure 4, the expression of NLRP3, ASC, pro‐caspase‐1, and Caspase‐1 was significantly elevated in the OGD/R group comparing with the control group, while PM2.5 further increased the levels of these proteins (Figure 4A,B). Consistently, the ELISA results showed that the production of inflammatory cytokines IL‐1β and IL‐18 was upregulated after OGD/R, and PM2.5 exposure further increased the expression of these two inflammatory factors (Figure 4C,D). Then, a Caspase‐1 activity assay was applied to evaluate the activity of Caspase‐1 in HMC‐3 cells after exposed to PM2.5 under ischemic state. Notably, significant activation of Caspase‐1 was observed in the OGD/R model, and Caspase‐1 activation was further enhanced after PM2.5 exposure (Figure 4E). These results indicated that PM2.5 exposure significantly induced NLRP3 inflammasome activation under ischemic conditions.

### 3.4 PM2.5 exposure induced GSDMD‐mediated pyroptosis

**FIGURE 5:**
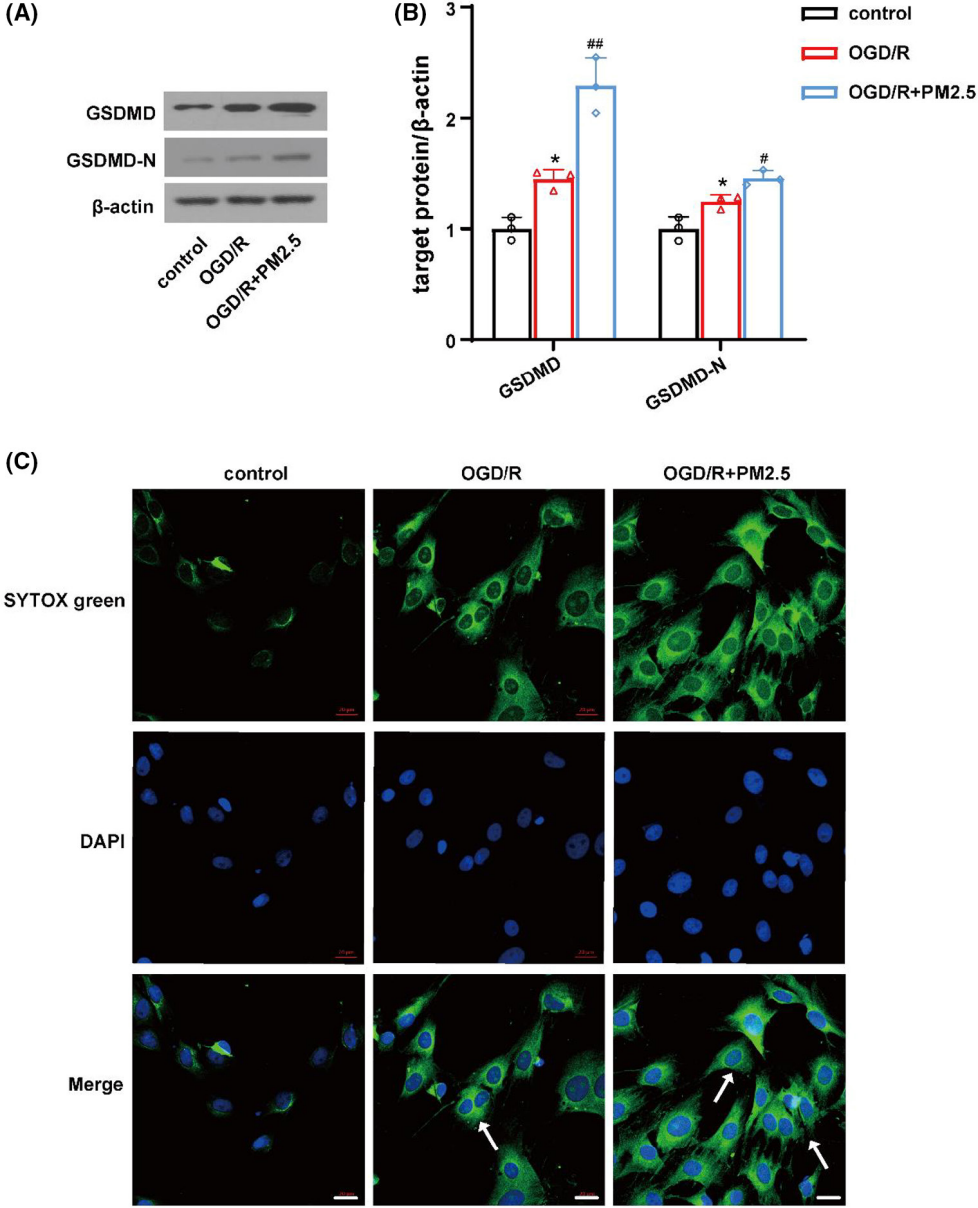
PM2.5 exposure induced cell pyroptosis under ischemic stress in vitro. (A, B) The expressions of GSDMD and GSDMD‐N in control, OGD/R, and OGD/R+PM2.5 groups (^*^ *p* < 0.05 vs. control; *^#^p* < 0.05, *^##^p* < 0.01 vs. OGD/R; *n* = 3). (c) The confocal immunofluorescence staining of DAPI and SYTOX was detected in the three groups. Scale bar = 20 μm

To investigate whether pyroptosis is involved in PM2.5‐induced ischemic injury, we assessed the expression and cleavage of GSDMD by immunoblotting. The protein levels of GSDMD and GSDMD‐N were significantly elevated after OGD/R, while PM2.5 exposure further increased the levels of the two proteins (Figure 5A,B). Subsequently, the confocal immunofluorescence staining of DAPI and SYTOX was used to assess the membrane damage and cell death. DAPI, a nuclear dye with blue fluorescence, is permeable to all cells and stains the nuclear content of all cells. SYTOX green, a nuclear dye with green fluorescence, is impermeable and enters the cells only in case of damage to the membrane and used to assess pyroptosis.^32^ Hence, its presence inside the HMC‐3 cells is an indication of plasma membrane rupture and leakage of cell contents. We observed the number of SYTOX green‐positive cells increased in HMC‐3 cells following OGD/R, while PM2.5 exposure further increased the SYTOX green uptake (Figure 5C). Collectively, these data suggested that GSDMD‐induced pyroptosis was further activated in vitro after PM2.5 exposure under ischemic state.

### 3.5 NLRP3 inhibition attenuated the effects of PM2.5 on NLRP3 inflammasome activation and pyroptosis

**FIGURE 6:**
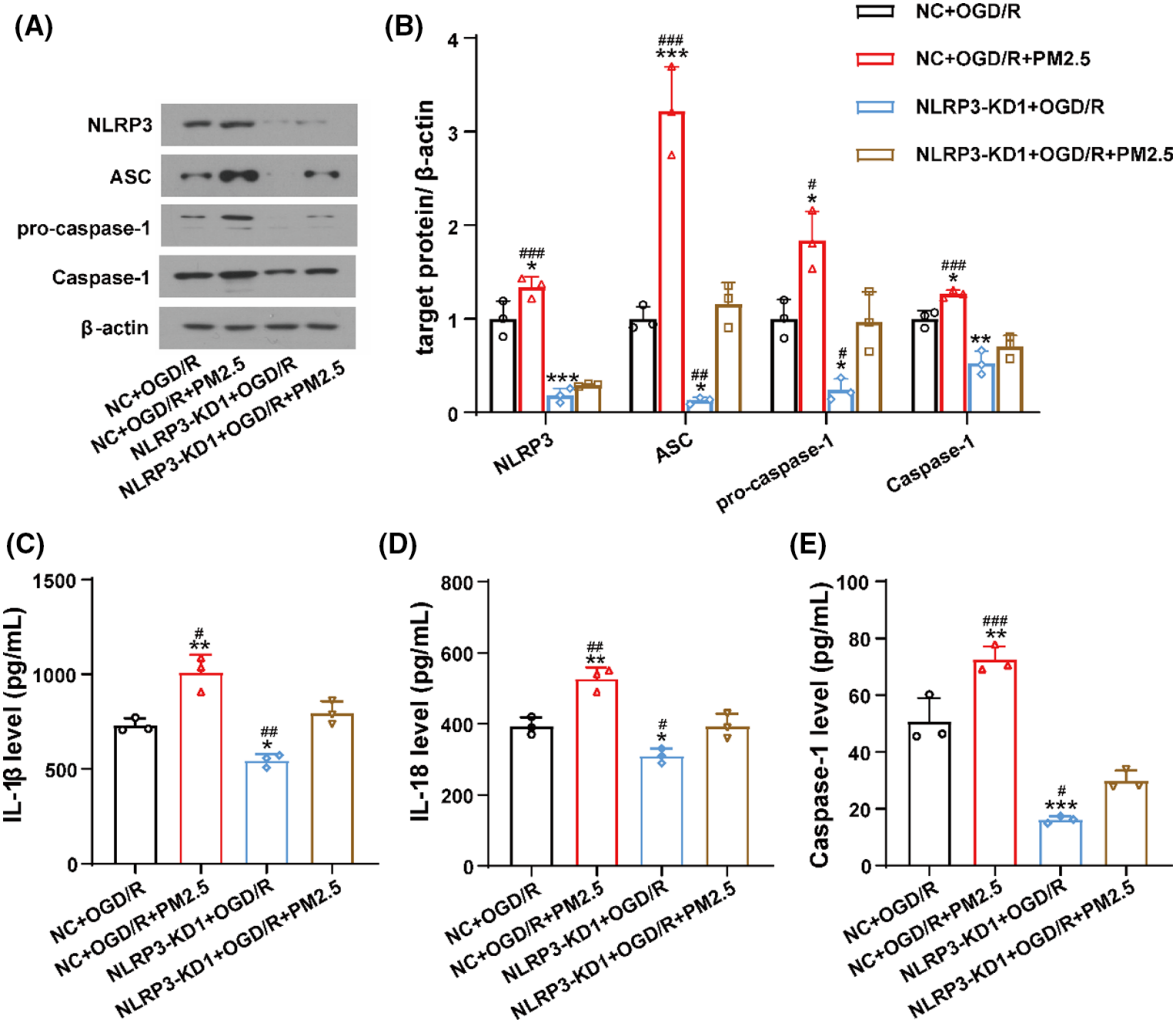
NLRP3 inhibition attenuated the effects of PM2.5 on NLRP3 inflammasome activation. (A, B) The expressions of NLRP3, ASC, pro‐caspase‐1, and Caspase‐1 of HMC‐3 cells in NC+OGD/R, NC+OGD/R+PM2.5, NLRP3‐KD1+OGD/R, and NLRP3‐KD1+OGD/R+PM2.5 groups. (C, D) The ELISA showed the production of inflammatory cytokines IL‐1β and IL‐18 in four groups. (E) Caspase‐1 activity was assessed in four groups (^*^ *p* < 0.05, ^**^ *p* < 0.01, ^***^ *p* < 0.001 vs. NC+OGD/R; *^#^p* < 0.05, *^##^p* < 0.01, *^###^p* < 0.001 vs. NLRP3‐KD1+OGD/R+PM2.5, *n* = 3)

To further validate the effects of PM2.5 on the NLRP3 inflammasome during ischemic stroke, we constructed a NLRP3‐KD1 HMC‐3 cell line and subjected the cells to OGD/R with or without PM2.5 exposure. As expected, NLRP3, ASC, pro‐caspase‐1, and Caspase‐1 expressions were significantly decreased in the NLRP3‐KD1 group when compared to the NC+OGD/R group (Figure 6A,B). Accordingly, the production of IL‐1β and IL‐18 and the activity of Caspase‐1 were suppressed when NLRP3 was knocked down (Figure 6C–E). PM2.5 exposure significantly increased the expression of these proteins, and enhanced the production of IL‐1β and IL‐18 and activity of Caspase‐1. However, the effects of PM2.5 on the NLRP3 inflammasome after OGD/R were significantly mitigated after NLRP3 was knocked down, as the expression of NLRP3, ASC, pro‐caspase‐1, and Caspase‐1 was suppressed (Figure 6A,B), accompanied by the downregulation of IL‐1β, IL‐18, and Caspase‐1 (Figure 6C–E).

**FIGURE 7:**
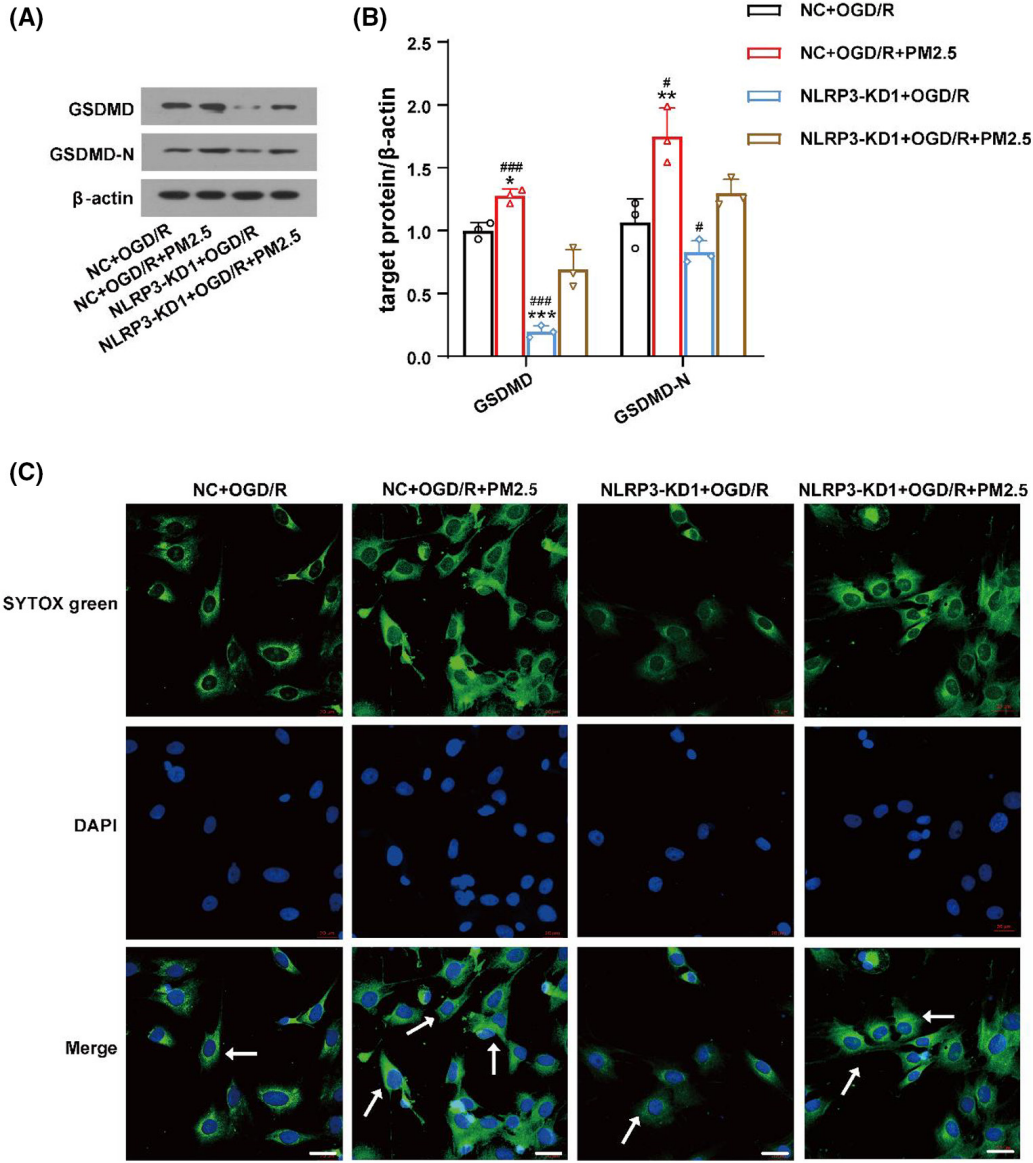
NLRP3 inhibition attenuated the effects of PM2.5 on cell pyroptosis. (A, B) The expressions of GSDMD and GSDMD‐N in NC+OGD/R, NC+OGD/R+PM2.5, NLRP3‐KD1+OGD/R, and NLRP3‐KD1+OGD/R+PM2.5 groups (*p<0.05, **p<0.01, ***p<0.001 vs. NC+OGD/R; *^#^p*<0.05, *^###^p*<0.001 vs. NLRP3‐KD1+OGD/R+PM2.5; *n* = 3). (C) The confocal immunofluorescence staining of DAPI and SYTOX was detected in the four groups in HMC‐3 cells. Scale bar = 20 μm

To determine the effects of PM2.5 on pyroptosis after ischemic stroke, the expression of GSDMD and GSDMD‐N was detected in NC+OGD/R, NC+OGD/R+PM2.5, NLRP3‐KD1+OGD/R, and NLRP3‐KD1+OGD/R+PM2.5 groups. As shown in Figure 7, the level of GSDMD and GSDMD‐N was significantly elevated after PM2.5 exposure, while NLRP3 depletion attenuated the effects of PM2.5 on the expression of these two proteins (Figure 7A,B). In addition, the immunofluorescence staining showed the number of SYTOX green‐positive cells decreased after NLRP3‐KD1 treatment following OGD/R. PM2.5 exposure promoted the SYTOX uptake, but the effect was attenuated when NLRP3 was knocked down (Figure 7C).

Interestingly, we observed that when comparing to the NLRP3‐KD1+OGD/R group, PM2.5 exposure still can increase the expression of ASC, pro‐caspase‐1 (Figure 6A,B), GSDMD and GSDMD‐N (Figure 7A,B) to some extent even though NLRP3 was depleted, suggesting that PM2.5 may induce NLRP3 inflammasome activation and pyroptosis via multiple mechanisms.

Together, these data provided further evidence that PM2.5 exposure induced the activation of NLRP3 inflammasome and pyroptosis, as suppressing NLRP3 notably reversed the enhancing effects of PM2.5 on the NLRP3 inflammasome and pyroptosis under ischemic stress.

### 3.6 PM2.5 exposure aggravated NLRP3 inflammasome activation by increasing ROS production following OGD/R

**FIGURE 8:**
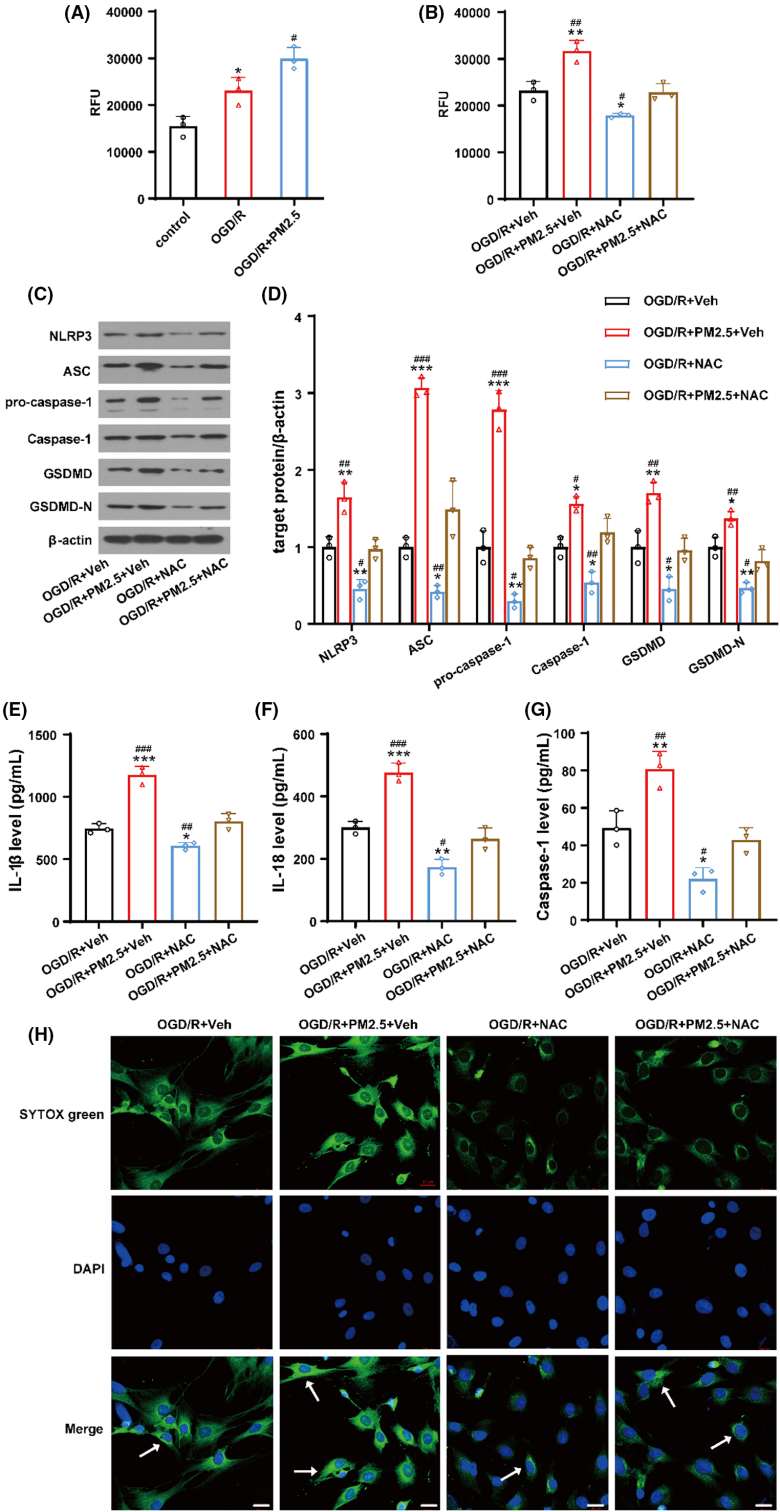
Inhibiting ROS production with NAC partially attenuated PM2.5‐induced NLRP3 inflammasome activation and pyroptosis under ischemic conditions. (A) The production of ROS in control, OGD/R and OGD/R+PM2.5 groups (^*^ *p* < 0.05 vs. control; *^#^p* < 0.05 vs. OGD/R; n = 3). (B) The production of ROS in OGD/R+Veh, OGD/R+PM2.5+Veh, OGD/R+NAC, and OGD/R+PM2.5+NAC groups. (C, D) The expressions of NLRP3, ASC, pro‐caspase‐1, Caspase‐1, GSDMD, and GSDMD‐N in four groups. (E, F) The production of inflammatory cytokines IL‐1β and IL‐18 in four groups. (G) Caspase‐1 activity was assessed in four groups (*p < 0.05, **p < 0.01, ***p < 0.001 vs. OGD/R+Veh; *^#^p* < 0.05, *^##^p* < 0.01, *^###^p* < 0.001 vs. OGD/R+PM2.5+NAC; *n* = 3). (H) The detection of DAPI and SYTOX green‐positive cells in the four groups. Scale bar = 20 μm

Oxidative stress has been reported to contribute to the toxicological mechanism of PM2.5, causing negative health effects.^33^ Some studies have proven that ROS can induce NLRP3 inflammasome activation after ischemic stroke.^34^ In order to explore whether ROS are involved in PM2.5‐induced NLRP3 inflammasome activation and pyroptosis after OGD/R, we measured intracellular ROS levels in HMC‐3 cells exposed with or without PM2.5 under ischemic conditions. As depicted in Figure 8A, the production of ROS markedly increased in the OGD/R group and was further increased after PM2.5 exposure. Administration of NAC, an ROS inhibitor, used 2 h before PM2.5 exposure, significantly decreased the production of ROS in OGD‐treated cells in both the presence and the absence of PM2.5 (Figure 8B).

Afterwards, we assessed the expression of NLRP3 inflammasome components and pyroptosis in OGD/R+Veh, OGD/R+PM2.5+Veh, OGD/R+NAC, and OGD/R+PM2.5+NAC groups. As shown in Figure 8, NAC treatment decreased the expression of NLRP3, ASC, pro‐caspase‐1, and Caspase‐1 following OGD/R (Figure 8C,D). The production of IL‐1β and IL‐18 and the activity of Caspase‐1 were suppressed after NAC treatment (Figure 8E–G). Pyroptosis was also inhibited by NAC, as the expression of GSDMD and GSDMD‐N (Figure 8C,D) and the number of SYTOX green‐positive cells (Figure 8H) were decreased under ischemic state. After PM2.5 exposure, the NLRP3 inflammasome was activated and pyroptosis was enhanced when compared to the OGD/R+Veh group. However, the effects of PM2.5 on NLRP3 inflammasome activation and pyroptosis were attenuated by NAC, as the expression of all the proteins decreased (Figure 8C,D). Furthermore, NAC treatment decreased the production of IL‐1β and IL‐18, suppressed the activity of Caspase‐1, and decreased SYTOX green‐positive cells when compared to the OGD/R+PM2.5+Veh group (Figure 8E–H). Taken together, these results suggested that PM2.5 exposure may enhance the NLRP3 inflammasome activation and pyroptosis by increasing OGD‐induced ROS production.

## 4 DISCUSSION

PM2.5 is one of the important constituents of air pollution and causes significant damage to human health. The chronic toxic effect of PM2.5 leading to ischemic stroke has also been gradually recognized in recent years. It is reported that exposure to PM2.5 can induce acceleration of atherosclerosis, alter vasomotor tone, cause vascular inflammation, and promote blood coagulation,^35^, ^36^, ^37^ contributing to the development of ischemic stroke. However, the underlying mechanisms are not understood yet. In this study, we observed that PM2.5 exposure decreased cell viability in a dose‐ and time‐dependent manner in BV‐2 and HMC‐3 cells under ischemic conditions. Then, we found that PM2.5 exposure induced NLRP3 inflammasome activation and pyroptosis after OGD/R, which was attenuated when NLRP3 was knocked down. Furthermore, we discovered that inhibiting intracellular ROS production attenuated PM2.5‐induced NLRP3 inflammasome activation and pyroptosis following OGD/R, providing novel evidence that PM2.5 may aggravate ischemic injury by triggering NLRP3 inflammasome activation and pyroptosis.

To date, many epidemiologic studies have shown that exposure to PM2.5 increases both the incidence and mortality of ischemic stroke. A five‐year follow‐up study revealed an association between PM2.5 exposure and hospital admissions for stroke.^38^ The researchers showed that when PM2.5 concentration increased by 10 μg/ml,^3^ the risk of emergency hospital admissions for stroke causes increased by 1.29%. A meta‐analysis comprising 11 cohorts in Europe suggested that the risk of incident stroke increases 19% for every 5 mg/m^3^ increment in PM2.5 exposure.^39^ Over a mean follow‐up of 9.4 years, Qiu et al.^40^ confirmed that long‐term PM2.5 exposure was associated with a higher risk of incident ischemic stroke, but its association with incident hemorrhagic stroke was less clear. Furthermore, Wilker et al.^41^ demonstrated that exposure to PM2.5 was associated with smaller total cerebral brain volume and higher risk of covert brain infarcts, suggesting that PM2.5 has insidious effects on structural brain aging, even in dementia‐ and stroke‐free persons. In the rodent study, Zhang et al.^42^ reported that PM2.5 exposure induced behavioral changes and neurological deficits in middle cerebral artery occlusion (MCAO) rats. In the present study, we established an OGD/R model and found that exposure to PM2.5 significantly reduced the viability of microglial cells in a dose‐dependent manner, which was in agreement with the previous study showing PM2.5 caused cell cycle arrest and the cell proliferation inhibition in neuronal cells.^43^ However, the biological mechanisms linking PM2.5 exposure with damage to the cerebrovascular system remain unclear.

Neuroinflammation associated with microglia has been identified as a major contributor to ischemic stroke pathogenesis.^44^ The inflammasome detects pathogen infections, tissue damage, or metabolic imbalances, leading to the maturation and release of proinflammatory cytokines, which participate in inflammatory reactions.^45^ A number of studies have highlighted the role of the NLRP3 inflammasome, which is fundamental to the innate immune system and contributes to neuronal and glial cell death during ischemic stroke. In 2013, Fann et al.^22^ discovered that the NLRP3 inflammasomes were activated with increased levels of NLRP3, ASC, Caspase‐1, and both IL‐1β and IL‐18 in mice under ischemic conditions in vivo and vitro. Yang et al.^23^ further evidenced that NLRP3^−/−^ mice subjected to MCAO exhibited a reduced infarct volume, decreased edema formation, and preservation of blood–brain barrier (BBB) permeability. A recent study showed that the NLRP3 inflammasome in neurons drives neuroinflammation in ischemic stroke, while blocking NLRP3 protected against ischemia/reperfusion injury by mitigating inflammation and stabilizing the BBB.^46^ Furthermore, some studies have suggested that the NLRP3 gene polymorphisms are related to the occurrence of ischemic stroke, providing significant clinical evidence linking NLRP3 and the risk of ischemic stroke.^47^, ^48^ Consistent with previous reports,^22^, ^24^ we found that the NLRP3 inflammasome was activated in HMC‐3 cells following OGD/R and that the expression of NLRP3 inflammasome components was elevated, Caspase‐1 activity was enhanced, and IL‐1β and IL‐18 production was elevated. However, shRNA NLRP3 treatment reversed these effects and inhibited NLRP3 inflammasome activation after OGD/R. In addition, the results of the CCK‐8 assay indicated that cell proliferation was increased in the groups in which NLRP3 was inhibited with shRNAs compared to the OGD‐alone group on different days. These data further proved that inhibiting NLRP3 inflammasome activation may attenuate ischemic injury in vitro.

PM pollution has been reported to be associated with neuroinflammation in the brain. Morgan et al.^49^ discovered that chronic nanoscale PM induced microglial activation, enhanced inflammatory responses, and upregulated IL‐1α and tumor necrosis factor‐α (TNF‐α). Guo et al.^50^ reported that PM10 exposure upregulated inducible nitric oxide synthase (iNOS), IL‐1β, and intercellular adhesion molecule‐1 (ICAM), caused endothelial dysfunction and inflammatory response, and increased the risk of ischemia‐like injuries in a season‐dependent manner in rats. A recent study revealed that PM2.5 increased the production of nitric oxide and ROS and upregulated the transcription of various proinflammatory markers in BV‐2 cells, leading to neurotoxicity and cell death.^51^ Additionally, another study demonstrated that PM2.5 exposure increased the levels of IL‐6, IL‐1β, and TNF‐α and induced neuroinflammation in the cortex, hippocampus, and striatum.^52^ Till now, accumulating evidence has suggested that PM2.5 exposure can induce NLRP3 inflammasome activation in airway epithelial cells, ventricular myocytes, corneal epithelial cells, and epidermal cells, causing toxic effects and aggravating lung injury, cardiovascular injury, corneal damage, and skin inflammation.^27^, ^28^, ^53^, ^54^ Moreover, some studies have demonstrated that PM2.5 exposure may aggravate neural injury and neuroinflammation in AD through NLRP3 inflammasome activation.^29^, ^55^ However, the relationship between PM2.5 and NLRP3 inflammasome activation during ischemic stroke has rarely been explored. In the present study, we found that PM2.5 exposure upregulated the expression of NLRP3 inflammasome components that may facilitate NLRP3 inflammasome assembly, which then increased the production of IL‐1β and IL‐18, potentially inducing neuroinflammation and aggravating ischemic injury. To verify the relationship between PM2.5 exposure and the NLRP3 inflammasome during ischemic stroke, we carried out shRNA‐mediated knockdown of NLRP3 in HMC‐3 cells under ischemic conditions. The CCK‐8 assay showed that NLRP3 depletion increased the viability of BV‐2 and HMC‐3 cells on different days after PM2.5 exposure. More importantly, inhibiting NLRP3 attenuated the effects of PM2.5 exposure on NLRP3 inflammasome activation after OGD/R, decreasing the expression of NLRP3 components, and suppressing inflammatory factor production and Caspase‐1 activity. Our data further provide evidence that PM2.5 may induce NLRP3 inflammasome activation and aggravate neuroinflammation in vitro under ischemic stress.

Pyroptosis, as a pro‐inflammatory form of lytic cell death, can be triggered by various pathological stimuli, including microbial infection, heart attack, cancer, and brain injury.^56^, ^57^, ^58^, ^59^ It is accompanied by plasma membrane rupture, water influx, cellular swelling, osmotic lysis, and the release of proinflammatory cellular content.^20^ Zhong et al.^60^ reported that instillation of PM2.5 suspension for five days induced cardiac and lung inflammatory injuries and increased inflammasome‐mediated pyroptosis in mice. In 2021, Li et al. found that PM2.5 exposure can lead to ocular hypertension and glaucoma by inducing cell pyroptosis and inflammation in intraocular tissues.^61^ Consistently, Niu et al.^53^ demonstrated that PM2.5 exposure triggered corneal inflammation and pyroptosis via NLRP3 activation, contributing to corneal damage. In this study, we found that pyroptosis was induced following OGD/R, accompanied by the upregulation of GSDMD and GSDMD‐N and the elevation of the number of SYTOX green‐positive cells. Then, we found that PM2.5 exposure further enhanced pyroptosis under ischemic conditions, while inhibiting NLRP3 mitigated the effects of PM2.5 exposure on pyroptosis, further supporting the notion that the toxic effects of PM2.5 on brain health may be associated with NLRP3 inflammasome and GSDMD‐mediated pyroptosis.

To date, the exact mechanism and cellular stimuli leading to NLRP3 inflammasome activation are poorly understood. There is emerging evidence indicating that the NLRP3 inflammasome can be activated by three major models: K^+^ efflux out of the cell, ROS production, and cathepsin release after lysosomal destabilization.^12^ An increasing number of studies have linked oxidative stress to NLRP3 inflammasome activation during ischemic stroke.^23^ ROS are proposed to serve as upstream signals mediating NLRP3 inflammasome activation and stroke‐induced neuroinflammation.^62^ In addition, oxidative stress has also been proven to be involved in the toxic mechanism of PM2.5.^63^ In the current study, we showed that intracellular ROS levels were significantly increased in HMC‐3 cells subjected to OGD/R, while PM2.5 exposure further increased ROS production. Then we used NAC, an ROS scavenger, to investigate the role of ROS in NLRP3 inflammasome activation and pyroptosis after PM2.5 exposure. We found that NAC suppressed NLRP3 inflammasome activation, decreased Caspase‐1 activity, and reduced the production of IL‐1β and IL‐18 in PM2.5‐OGD‐treated cells, suggesting that inhibiting the production of ROS may attenuate the effects of PM2.5 exposure on NLRP3 inflammasome activation under ischemic state. Furthermore, NAC suppressed PM2.5‐induced pyroptosis, as NAC downregulated the expression of GSDMD and GSDMD‐N and decreased the number of SYTOX green‐positive cells. These data indicated that ROS production is a crucial event in NLRP3 inflammasome processing and pyroptosis in response to PM2.5 during ischemic stroke.

In addition to ROS produced intrinsically, the nicotinamide adenine dinucleotide phosphate (NADPH) oxidase pathway and the damaged mitochondria also lead to intracellular ROS production. However, we did not use other ROS scavenger such as NADPH oxidase inhibitor diphenylene iodonium (DPI), or mitochondria‐targeted antioxidant MitoQ, to explore the source of ROS. More studies are needed to investigate the molecular mechanisms of ROS and NLRP3 inflammasome activation in PM2.5‐induced neuronal injury during ischemic stroke.

## 5 CONCLUSION

In the present study, we demonstrated that PM2.5 exposure triggered the activation of the NLRP3 inflammasome and pyroptosis under ischemic conditions, which may be mediated by increased ROS production after ischemic stroke. The findings of the present study provide a more enhanced understanding of the interplay between PM2.5 and neuroinflammation and cell death, and reveal a novel mechanism of PM2.5‐mediated suppression of intracellular inflammatory responses after ischemic stroke. These findings may be meaningful for both environmental and health policy developments related to air pollution and stroke prevention.

## CONFLICT OF INTEREST

All of the authors declare no conflict of interest.

## AUTHOR CONTRIBUTIONS

Li Gao, Jie‐Xing Qin, Yang‐Tai Guan, and Qing Dong conceived, designed, and coordinated the study. Li Gao, Jie‐Xing Qin, Jian‐Quan Shi, and Teng Jiang performed the experiments and analyzed the data. Fei Wang, Chong Xie, and Nan Zhi helped with cell culture and CCK‐8 assay. Qing Gao performed the qRT‐PCR. Li Gao, Jie‐Xing Qin, Yang‐Tai Guan, and Qing Dong wrote and revised the manuscript, and checked the data analysis. All the authors revised and approved the final version of the manuscript.

## Supporting information

Figure S1

## Data Availability

The original contributions presented in the study are included in the article/Supplementary Material, further inquiries can be directed to the corresponding author.
