# Supplementary figures and images for: Fine particulate matter exposure aggravates ischemic injury via NLRP3 inflammasome activation and pyroptosis
Source: CNS Neurosci Ther. 2022 Apr 10;28(7):1045–58. doi: 10.1111/cns.13837 (PMC9160454; doi:10.1111/cns.13837)

Sup. Figure 1


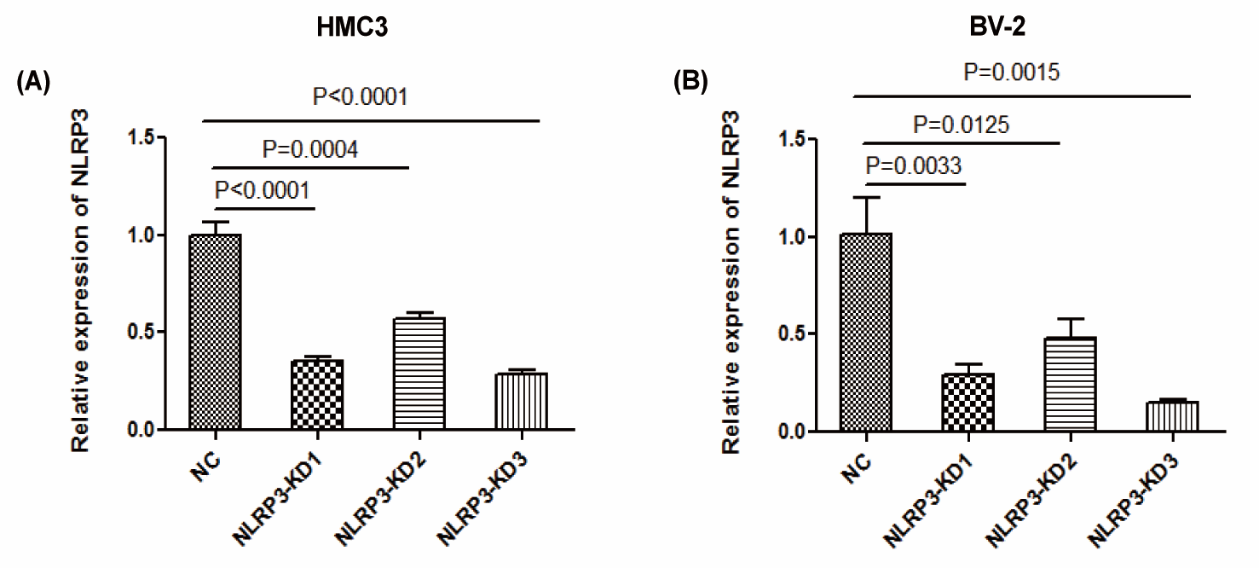

Supplement: Supplementary file 1 — Figure S1 [file CNS-28-1045-s001.docx]
